# A Pilot Study Comparing Postural Control in Adolescents With and Without Neurodevelopmental Disorders: Evaluation Under Open-Eyed, Closed-Eyed, and Auditory Stimulation Conditions

**DOI:** 10.7759/cureus.95706

**Published:** 2025-10-29

**Authors:** Masatoshi Maeshige, Tadayuki Iida, Atsuko Morikawa

**Affiliations:** 1 Iroha Home Nursing and Rehabilitation Station, Kanon Corporation, Hiroshima, JPN; 2 Physical Therapy, Faculty of Health and Welfare, Prefectural University of Hiroshima, Mihara, JPN; 3 Rehabilitation Medicine, Department of Research and Development (Think Tank Division) Kanon Corporation, Hiroshima, JPN

**Keywords:** adolescents with asd, auditory sensory processing, force plate measurement, neurodevelopmental disorders, static postural control

## Abstract

Background: This pilot study aimed to examine postural control characteristics in adolescents with neurodevelopmental disorders (NDDs), such as autism spectrum disorder (ASD), who often show atypical postural regulation due to sensory integration challenges. While sensory factors have received increasing attention, few studies have investigated postural sway under different environmental conditions, particularly auditory stimulation. The primary objective was to compare the center of pressure (COP) sway between adolescents with and without NDDs under open-eye, closed-eye, and auditory conditions. Secondary objectives included evaluating directional sway (X- and Y-axis) and the specific effects of auditory input.

Methods: This cross-sectional pilot study included nine adolescents with NDDs and 37 typically developing (TD) peers. Physical measurements (height, weight, BMI, joint laxity, grip strength, knee extension strength) and postural control were assessed. Static standing balance was measured barefoot for 30 seconds in three conditions (open eyes, closed eyes, auditory stimulation with a non-rhythmic piano composition) using a force plate. Each condition was measured once for 30 seconds. COP parameters included total path length, path length per unit area, and directional displacement. Between- and within-group comparisons were analyzed using the Mann-Whitney U and Wilcoxon signed-rank tests, with exact p-values, effect sizes (r), and 95% confidence intervals reported.

Results: Adolescents with NDDs exhibited significantly lower grip and knee extension strength than TD peers (p < 0.001, r = 0.47-0.68). COP sway was significantly greater in the NDD group under auditory stimulation (p < 0.001, r = 0.71). Within both groups, sway increased significantly under auditory stimulation compared to the open-eyes condition (p < 0.001, r = 0.25-0.52).

Conclusion: This pilot study provides preliminary evidence that adolescents with NDDs demonstrate increased postural sway under auditory stimulation, which may reflect sensory integration difficulties and reduced muscular control. Given the small and heterogeneous sample, these findings should be interpreted with caution. Larger and more homogeneous studies are needed to confirm the observed associations and to clarify the role of auditory input in postural stability among youth with developmental disorders.

## Introduction

Neurodevelopmental disorders (NDDs), as classified in the Diagnostic and Statistical Manual of Mental Disorders, Fifth Edition (DSM-5), encompass conditions such as autism spectrum disorder (ASD), attention-deficit/hyperactivity disorder (ADHD), developmental coordination disorder (DCD), and specific learning disorders (SLD) [1]. Among children with ASD, comorbidity with other NDDs is frequent-63.2% have coexisting DCD, and 50.6% also present with ADHD [2]. The prevalence of DCD alone is estimated at 5-6% in school-aged children, with a male-to-female ratio ranging from 3:1 to 7:1 [3].

Children and adolescents with NDDs often exhibit impaired postural control due to deficits in sensory integration. These impairments can manifest as poor posture, clumsiness, and limited motor coordination, contributing to decreased physical activity, social withdrawal, and lower self-esteem [4-6]. Previous studies have shown that individuals with ASD or DCD have greater postural sway than typically developing (TD) peers, especially when visual input is limited [7,8]. Additionally, recent physiological profiling has revealed distinctive postural sway patterns in children and adolescents with ASD compared to their typically developing peers [9]. Auditory input also influences balance in children with ASD; however, its effects vary depending on the type, timing, and predictability of the auditory stimulus [9,10].

Recent studies have examined how specific forms of auditory stimulation - such as rhythmic cueing or verbal encouragement - can impact motor behavior and postural stability. For example, El Shemy and El-Sayed (2018) reported that rhythmic auditory cueing improved gross motor function in children with ASD [11], while others have found that non-rhythmic or irregular auditory feedback alters postural control strategies in adolescents [12]. These findings underscore the need to consider auditory environments in rehabilitation and educational planning.

Despite growing interest in auditory-sensory interaction, few studies have directly compared the postural responses of adolescents with and without NDDs under auditory stimulation. Adolescence represents a key developmental stage during which postural and sensory systems continue to mature [13], yet this population remains underrepresented in postural control research. Additionally, the type of auditory stimulus may influence outcomes; non-rhythmic sounds with unpredictable temporal structures may better mimic real-world sensory environments and reduce bias from musical familiarity [14].

The present study aimed to compare static postural control in adolescents with and without NDDs under three conditions: open eyes, closed eyes, and auditory stimulation using a non-rhythmic piano composition. By analyzing center of pressure (COP) sway using a force plate, we sought to elucidate whether auditory input exacerbates postural instability in adolescents with NDDs. We hypothesized that postural sway would be significantly greater in the NDD group, particularly under auditory stimulation, reflecting challenges in sensory integration and postural regulation.

Given the exploratory nature and limited sample size of the current dataset, this investigation is positioned as a pilot study to generate preliminary evidence and inform future large-scale trials.

## Materials and methods

Participants

The participants were 37 TD children who belonged to a local soccer team (mean age: 13.5 ± 0.5 years) and nine children with NDDs attending after-school daycare (mean age: 12.5 ± 0.5 years). Diagnoses were made by physicians based on DSM-5 criteria, primarily through clinical judgment. In some cases, standardized assessments such as the WISC-IV were available, but instruments such as ADOS-2 or Conners’ scales were not systematically applied. Diagnoses included four with pervasive developmental disorder (PDD), three with autism spectrum disorder (ASD), one with ASD and intellectual disability (ID), and one with attention-deficit/hyperactivity disorder (ADHD) and ASD. Children with selective mutism were excluded, as this condition is not classified as an NDD according to DSM-5.

All participants had no history of surgical procedures involving the feet, ankles, knees, buttocks, back, brain, spinal cord, or inner ear. Exclusion criteria included severe disease history, orthopedic disorders, mental disorders other than NDDs, use of drugs that affect motor function, inability to understand test instructions, or lack of appropriate informed consent. Table 1 presents the diagnoses and intelligence test results for the NDD group.

The contents and methods of the study were explained in advance to both the participants and their guardians. The study was conducted after obtaining written informed consent from the guardians and assent from the children in a developmentally appropriate manner, in accordance with institutional policy. The study period was from April 1, 2020, to December 31, 2022, and took place in the conference room of the Iroha Home Nursing and Rehabilitation Station. This study complied with the Declaration of Helsinki and was approved by the institutional review board of Prefectural University of Hiroshima Mihara Campus (Approval No.: 19MH054). Due to recruitment feasibility and practical constraints, the sample sizes for the TD and NDD groups were not matched. Accordingly, the study was designed and reported as a pilot investigation.

**Table 1:** Diagnoses and intelligence test information in the neurodevelopmental disorders group ^*^ Wechsler Intelligence Scale for Children–Fourth Edition [15]; ^†^ Kaufman Assessment Battery for Children–Second Edition [16] Abbreviations: PDD, pervasive developmental disorders; ASD, autism spectrum disorder; ADHD, attention deficit hyperactivity disorder; ID, intellectual disability; FSIQ, full-scale intelligence quotient; VCI, verbal comprehension index; PRI, perceptual reasoning index.

Participants (n=9)	Diagnosis	FSIQ	VCI	PRI	Notes
A	PDD	No information	No information	No information	No handbook
B	PDD	No information	No information	No information	K-ABCⅡ^†^, cognitive comprehensive scale:90
C	ASD	No information	No information	No information	
D	ASD	71	69	85	WISC-Ⅳ, 2018
E	ASD	97	109	102	WISC-Ⅳ, 2019
F	ADHD・ASD	114	103	113	WISC-Ⅳ, 2015
G	PDD	80	93	100	WISC-Ⅳ, 2018
H	PDD	No information	No information	No information	
I	ASD・ID	69	68	78	WISC-Ⅳ, 2018

Physical measurements and strength

Physical measurements included height, weight, body mass index (BMI), circumference (upper arm, thigh, lower leg), and joint laxity. Muscle strength measurements included grip strength and knee extension strength. Measurements were conducted in the following order: height, weight, circumference, joint laxity, grip strength, and knee extension strength. A five-minute rest interval was provided before the postural sway assessment.

Height was measured with a measuring tape fixed against a wall. Body weight was measured using a digital scale. BMI was calculated as weight (kg) ÷ height² (m²). According to the World Health Organization (WHO), a BMI ≥ 30 is considered obese, while according to the Japanese Society for the Study of Obesity, a BMI < 18.5 is underweight, 18.5-24.9 is normal, and ≥ 25 is obese, further classified into obesity grades 1-4 [17].

Circumference was measured at standardized bony landmarks: the midpoint between the acromion and olecranon for the upper arm, the midpoint between the anterior superior iliac spine and the superior border of the patella for the thigh, and the midpoint between the fibular head and the lateral malleolus for the lower leg [18].

Joint laxity was assessed using the Tokyo University method, a standardized procedure widely applied in Japan. This method evaluates generalized joint hypermobility based on seven maneuvers: the ability of the thumb to touch the forearm during wrist flexion, hyperextension of the elbow joint beyond 15°, the ability to clasp the fingers behind the back on both sides, hyperextension of the knee joint beyond 10°, dorsiflexion of the ankle joint beyond 45°, forward flexion of the trunk in standing with the palms placed flat on the floor, and the ability to abduct the legs beyond 180° in a standing position. Each maneuver was scored as 1 point for a positive response, 0.5 points for asymmetry, and 0 points for a negative response. A total score ≥ 4 indicated generalized joint laxity [19].

Grip strength was measured using a digital dynamometer (T.K.K.5401, TAKEI Scientific Instruments Co., Ltd., Niigata, Japan) [20,21]. Participants stood with feet shoulder-width apart and arms hanging at their sides. The grip span was adjusted so that the proximal interphalangeal joint of the index finger formed a 90° angle. Participants were instructed to squeeze the device with maximal effort without touching their body or clothing. Measurements were taken twice per hand, alternating sides, and the maximum value was adopted. Results were expressed in kilograms, rounded down to the nearest whole number.

Knee extensor strength was measured using a handheld dynamometer with belt fixation (μTas F-1, ANIMA Corporation, Chofu City, Tokyo). Participants sat with hips and knees at 90° flexion, with a folded towel under the distal thigh. The sensor was attached to the ankle and fixed to the bed frame with a belt. Participants exerted maximal isometric effort for 3-5 seconds with verbal encouragement. Measurements were taken twice per side, and the larger value was adopted. Strength was expressed relative to body weight (Nm/kg), with decimal places rounded down.

Center of pressure (COP) sway in static standing

**Figure 1:**
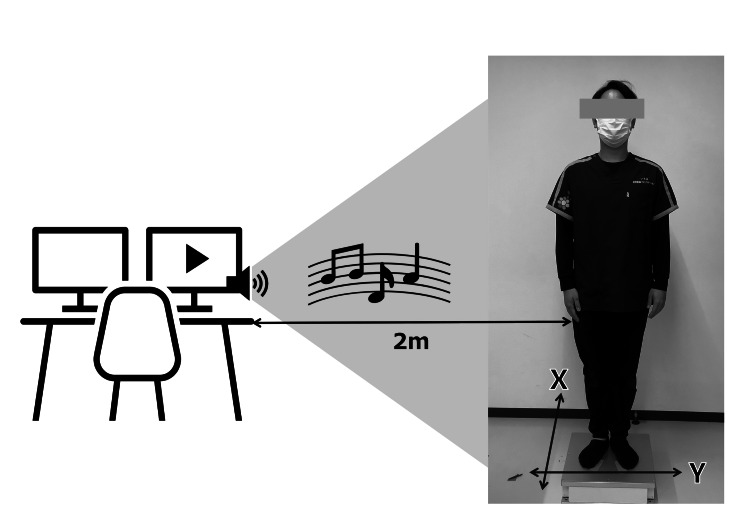
Measurement of center of pressure sway in the standing position using a force plate

Static standing balance was measured under three conditions: open eyes, closed eyes, and auditory stimulation with eyes open, using a force plate (SS-FP40AO, Sports Sensing Co., Ltd.) (Figure 1). Postural sway assessments were performed on the same day as physical measurements, following a five-minute rest interval. The order of the open-eye and closed-eye conditions was randomized, all conditions were measured in a “comfortable posture,” and the auditory stimulation condition was always conducted last.

“Comfortable posture” was defined as upright standing with both arms relaxed at the sides, barefoot, and fixating on a target at eye level 2 m away. Participants were instructed to remain as still as possible for a single 30-second trial under each condition. The 30-second duration was chosen based on its established validity in pediatric balance research (e.g., Fournier et al., 2010) [7].

COP parameters included total locus length, unit locus length, and displacement along the X (anteroposterior) and Y (mediolateral) axes. Larger values indicated greater sway magnitude. The X- and Y-axis definitions followed the wiring orientation of the force plate (Figure 1).

Auditory stimulation consisted of an algorithmically generated non-rhythmic piano composition (“The World’s Ugliest Music,” by Richard Scott [22]), designed to eliminate rhythmic regularity and reduce bias from familiarity. The music was delivered via external speakers positioned 2 m to the right of the participant at ~65 dB. Participants were instructed to maintain an upright stance and keep their eyes open throughout the 30-second trial.

Sample size

A power analysis using G*Power (Heinrich-Heine-Universität Düsseldorf, Düsseldorf, Germany) was conducted based on anteroposterior sway values reported by Fournier et al. [7]. The analysis indicated that 10 participants per group would be required (α = 0.05, power = 95%). To account for attrition, the initial NDD target was set at 10. One participant was excluded due to a diagnosis of selective mutism, leaving nine in the NDD group and 37 in the TD group (total n = 46). Due to feasibility limitations, the TD and NDD group sizes were not matched, and this study is reported as a pilot investigation. Participants were informed that they could withdraw at any time.

Statistical analyses

Between-group comparisons of physical measures and COP parameters were conducted using the Mann-Whitney U test. Within-group comparisons (open eyes vs. closed eyes; open eyes vs. auditory stimulation) were conducted using the Wilcoxon signed-rank test. Analyses were performed using EZR version 1.42. Exact p-values were reported, along with effect sizes (r) and their 95% confidence intervals, following Mizumoto et al. (2008) [23]. Measurement reliability was confirmed with intraclass correlation coefficients (ICC ≥ 0.85).

## Results

The TD group demonstrated significantly higher grip strength (left: p < 0.001, r = 0.52; right: p < 0.001, r = 0.47) and knee extension strength (left: p < 0.001, r = 0.66; right: p < 0.001, r = 0.68) than the NDD group (Table 2). In line with Cohen’s benchmarks, these effect sizes ranged from medium to large.

**Table 2:** Physical measurement values in typically developing and neurodevelopmental disorder groups p-value: Mann-Whitney U test Abbreviations: TD, typically developing; NDD, neurodevelopmental disorder; SD, standard deviation; IQR, interquartile range; BMI, body mass index

Parameters		TD(n=37)		NDD(n=9)		p value
		Mean/ Median	SD IQR	Mean/ Median	SD IQR	
Age(years)		13.5	0.6	12.4	0.5	<0.001
Height(m)		1.61	0.08	1.6	0.16	0.155
Body weight(kg)		48.5	7.8	50.1	12.2	0.846
BMI(kg/m2)		18.7	2.1	20.4	4.4	0.893
Joint laxity (point)		1.2	0.5-1.5	1.6	0.5-2.0	0.528
Circumference (cm)	Left upper arm	22.8	21.0-23.5	25.2	23.0-29.0	0.057
	Right upper arm	22.9	21.5-23.5	25	22.5-29.5	0.147
	Left thigh	40.3	3.8	41.2	4.9	0.744
	Right thigh	40.5	3.6	41.7	5.2	0.57
	Left lower leg	33	3.7	33.2	3.7	0.945
	Right lower leg	33.1	3.8	32.9	3.7	0.679
Grip (kg)	Left grip	26.6	6.3	18.7	6	<0.001
	Right grip	29	6.8	19.6	4.6	<0.001
Knee extensor strength (kgf/kg)	Left knee extensor strength	0.74	0.15	0.45	0.08	<0.001
	Right knee extensor strength	0.8	0.15	0.48	0.07	<0.001

We further analyzed postural sway under two standing conditions - open eyes (comfortable posture) and auditory stimulation. For both groups, total locus length and locus length per unit area were calculated. Under the auditory stimulation condition, total locus length was significantly greater in the NDD group than in the TD group (p < 0.001, r = 0.71), indicating a large effect size (Table 3).

Within-group comparisons showed that in the TD group, total locus length increased significantly from open eyes to auditory stimulation (p < 0.001, r = 0.52). The NDD group also exhibited a significant increase (open eyes → auditory stimulation: p = 0.004, r = 0.25), although the effect size was small. By contrast, locus length per unit area did not differ significantly between the two conditions within either group (Table 3).

**Table 3:** Comparison of center of pressure (COP) sway values with open eyes (comfortable position) and those with auditory stimulation in the typically developing and neurodevelopmental disorder groups p-value: Mann-Whitney U-test ^†^ p<0.001, Wilcoxon's signed-rank sum test; ^‡ ^p=0.004, Wilcoxon's signed-rank sum test

Parameter		TD (n=37)^†^			NDD (n=9)^‡^			p value
		Median	25%	75%	Median	25%	75%	
Open eyes (comfortable)	Total locus length (cm)	74.2	55.7	65.9	62.6	48	70.4	0.957
	Locus length per unit area (cm/s)	41606.9	33012.3	47289.7	98805.5	26356.2	61062.6	1
Auditory stimulation condition (eyes open)^†^	Total locus length (cm)	819.4	716.9	913.1	5900.3	1553.2	10852.3	<0.001
	Locus length per unit area (cm/s)	76995.1	26134.1	57778.4	116082.3	52229.7	106348	0.04

Directional components were also examined along the X-axis (anteroposterior) and Y-axis (mediolateral). In the open-eyes condition, X-axis sway was significantly greater in the NDD group than in the TD group (p = 0.0078), suggesting increased anteroposterior instability in the NDD group, whereas no between-group difference was observed along the Y-axis under the same condition (Table 4). Under auditory stimulation, no significant between-group differences were detected for either axis. In within-group analyses, the TD group showed a significant increase in X-axis sway from open eyes to auditory stimulation (p < 0.001), while the NDD group did not reach statistical significance on this comparison (p = 0.164), despite a small elevation in the median value. Y-axis sway remained broadly unchanged across conditions in both groups (Table 4).

**Table 4:** Between- and within-group comparisons of directional center of pressure sway (X- and Y-axes) under eyes-open and auditory stimulation conditions p value: Mann-Whitney U-test ^†^ p<0.001, Wilcoxon's signed-rank sum test; ^‡ ^p=0.164, Wilcoxon's signed-rank sum test

Parameter		TD (n=37)^†^			NDD (n=9)^‡^			p value
		median	25%	75%	median	25%	75%	
Open eyes (comfortable)	Center of sway displacement in X (cm)	-0.121	-0.128	-0.112	0.081	0.119	0.035	0.0078
	Center of sway displacement in Y (cm)	0.01	-0.006	0.028	0.006	0.004	0.032	0.912
Open eyes (the sense of hearing)	Center of sway displacement in X (cm)	-2.349	-2.567	-913.1	8.102	-3.225	9.058	0.542
	Center of sway displacement in Y (cm)	0.016	-0.014	0.026	0.011	-0.017	0.015	0.658

## Discussion

In this pilot study, we found that adolescents with NDDs exhibited significantly greater total COP path length than their TD peers under auditory stimulation with piano sounds. This finding suggests that auditory input can exacerbate postural instability in NDD populations. One possible explanation involves the concept of sensory reweighting, whereby the relative contributions of visual, vestibular, and somatosensory inputs to postural control vary depending on individual characteristics and environmental demands [24]. Recent evidence indicates that vestibular dysfunction contributes to postural instability in children with ASD (Chisari et al., 2024) [5], supporting the hypothesis that atypical sensory integration underlies increased sway. In the present context, attentional resources that are normally distributed across visual, vestibular, and somatic channels during upright stance may have been partially reallocated to auditory input, thereby destabilizing posture.

Previous studies provide further support for this interpretation. Hannant et al. [25] reported that children with ASD demonstrate heightened auditory and visual sensitivities and that interactions between auditory and visual systems influence motor control through projections to the eyes and frontal motor regions. Park et al. [26] found that auditory frequency modulated COP sway, particularly in the anteroposterior direction, in young adults. Increases in sway magnitude were observed at frequencies both above and below 2,000 Hz, whereas sound pressure had no consistent effect. In light of these findings, it is possible that adolescents with NDDs may have heightened auditory sensitivity or hyperacusis, which could contribute to their greater sway under auditory stimulation. However, because auditory frequency was not manipulated in the present study, this interpretation remains speculative and requires further investigation. Additionally, Miller et al. [27] reported that reduced white matter integrity in ASD leads to delayed sensory transmission and inefficiencies in anticipatory and feedback mechanisms, potentially compounding difficulties in integrating multiple sensory inputs.

Directional sway analysis revealed that adolescents with NDDs displayed significantly greater anteroposterior sway (X-axis) than TD peers in the eyes-open condition. This may reflect fundamental sagittal plane instability related to atypical integration of visual and proprioceptive inputs. Moreover, while auditory stimulation significantly increased X-axis sway in the TD group, a similar but nonsignificant trend was observed in the NDD group. These findings suggest that auditory input may disrupt sagittal stability through attentional competition or reallocation in both groups, though effects in the NDD group were less pronounced. By contrast, mediolateral sway (Y-axis) did not differ significantly between groups or conditions, implying that lateral stability may be less sensitive to auditory perturbation during adolescence.

The importance of visual information for maintaining postural stability was reaffirmed in this study. Both groups exhibited significantly greater sway length with eyes closed than with eyes open, consistent with previous research [28,29]. This emphasizes the critical role of visual feedback in adolescent postural control and highlights its potential utility in clinical interventions. Interestingly, locus length per unit area did not differ significantly between eyes-open and eyes-closed conditions, suggesting that this parameter reflects fine reflexive control mediated primarily by proprioceptive inputs, with minimal modulation by vision [30]. These results align with prior observations that adolescents with ASD tend to rely more heavily on proprioceptive than visual information. Children with developmental coordination disorder (DCD) also show difficulties with cross-modal integration, including mismatches between visual and proprioceptive inputs (Asano, 2016 [31]; Bair et al., 2012 [32]). Such atypical sensory weighting and reduced adaptability to changing contexts may explain the broader instability observed in adolescents with NDDs.

In addition to sensory integration differences, group disparities in muscular strength must also be considered. The TD group, composed of soccer players, demonstrated significantly greater grip and knee extension strength, likely reflecting their higher baseline fitness and regular training. This difference in physical conditioning may have provided the TD group with greater resilience against postural instability. Thus, background factors such as fitness level and habitual activity should be acknowledged as potential confounders when interpreting between-group differences.

Taken together, these findings suggest that adolescents with NDDs have distinct postural control characteristics compared with TD peers. Their increased sway under auditory stimulation and eyes-closed conditions may reflect both atypical sensory integration and reduced muscular capacity. These results contribute preliminary evidence regarding the sensory and motor factors underlying postural control in NDDs, but must be interpreted cautiously given the small, heterogeneous sample and unequal group sizes.

Future directions

Future research should include larger and more diverse samples of adolescents with various NDDs (e.g., ASD, ADHD, DCD), and incorporate standardized diagnostic instruments. Controlling for confounders such as sex distribution, physical activity level, and comorbidities will improve the rigor of future analyses. Furthermore, systematic investigation of auditory frequency and modality effects on postural sway will be essential to clarify the role of auditory processing in balance control. Longitudinal and intervention studies may ultimately inform targeted therapeutic and educational strategies, including sensory-based interventions and tailored physical training to improve postural stability in youth with developmental disorders.

Limitations of the study

This study has several important limitations that should be acknowledged. First, the sample size was small and imbalanced, particularly in the NDD group (n = 9) compared with the TD group (n = 37). This discrepancy limits the statistical power and reduces the generalizability of the findings.

Second, participants in the TD group were primarily adolescent males actively engaged in soccer, whereas the NDD group included adolescents with varied neurodevelopmental diagnoses, mainly ASD but also ADHD and ID. These differences in physical activity background and diagnostic composition may have introduced bias into group comparisons of both postural control and muscle strength.

Third, although a priori power analysis was performed, the final NDD sample fell short due to the exclusion of one participant, highlighting the difficulty of recruitment in this population. In addition, diagnostic confirmation relied primarily on clinical judgment by physicians, and standardized instruments such as ADOS-2 or Conners’ scales were not uniformly applied.

Fourth, potential confounders such as age, sex distribution, and BMI were not matched or statistically adjusted between groups, which may have influenced the results. Furthermore, corrections for multiple comparisons (e.g., Bonferroni or false discovery rate) were not applied, and this should be considered when interpreting the statistical significance of the findings.

Despite these limitations, this pilot investigation provides preliminary insights into the effects of auditory stimulation on postural control in adolescents with NDDs. The study underscores the need for future research with larger, more diverse, and diagnostically stratified samples, along with more rigorous methodological control, to strengthen reproducibility and generalizability.

## Conclusions

In this cross-sectional pilot study, we examined differences in postural sway between adolescents with NDDs and their typically developing peers under three static standing conditions: open eyes, closed eyes, and auditory stimulation. Adolescents with NDDs demonstrated greater sway than TD adolescents, particularly under auditory stimulation and eyes-closed conditions.

These findings suggest that postural control in adolescents with NDDs may be more vulnerable to sensory perturbations, especially involving auditory and visual inputs. At the same time, differences in muscular strength between groups, likely influenced by fitness level, should also be considered when interpreting these results.

Given the small and heterogeneous sample and methodological constraints, the present study should be regarded as preliminary. Nevertheless, the findings provide a foundation for future investigations and may inform the development of clinical assessment and intervention strategies aimed at improving postural stability in youth with developmental disorders.
